# Supplementary material for: Adopting an American framework to optimize nursing admission documentation in an Australian health organization
Source: JAMIA Open. 2022 Jul 11;5(3):ooac054. doi: 10.1093/jamiaopen/ooac054 (PMC9272497; doi:10.1093/jamiaopen/ooac054)
Supplement: ooac054_Supplementary_Data [file ooac054_supplementary_data.docx]

**APPENDIX A**

Comparison of Cerner International ECD and the modified Australian ECD approaches

|  | **International ECD** | **Australian ECD** |
| --- | --- | --- |
| **PHASE 1**  ***Review of Data*** |  |  |
| Baseline metrics  ● Cerner data element  extract  ● eMR form timers  (time elapsed from form activation to form completion)  ● eMR Click counters  (number of clicks while  completing the form)  ● eMR explorer Adult Admission Assessment Powerform audit report | Yes  Yes  Yes  No | Yes  Not available  Not available  Yes |
| Quantitative Analysis  ● Data element utilization  rates  ● Time from activating form  to completing form  ● Number of clicks during  form completion  ● Time elapsed from  admission time to form completion time | Yes  Yes  Yes  No | Yes  Not available  Not available  Yes |
| **Phase 2**  ***Review of Practice*** |  |  |
| Approach | eMR audit reports | Consultations with nursing staff (Clinical Nurse Educators and managers) in the district about admission and AAA form |
| **Phase 3**  ***Review of Policy*** |  |  |
| Resources reviewed | USA Federal regulatory standards  Literature review | Australian National Standards  NSW Ministry of Health guidelines/policies  Consultation with district subject matter experts (Clinical Nurse Consultants) to provide national, state, or local district policies that specifically relate to data elements in the AAA form at point of admission  Relevant Australian and NSW health literature |
| **Phase 4**  ***ECD Workshop*** |  |  |
| ECD Collaborative workshop participants | Cerner USA  Chief Nursing Informatics Officers | Cerner Australia  Chief Nursing and Midwifery Information Officer  ECD Coordinator  Health Informatics teams Clinical Nurse Educators  eMR applications specialist |
| Duration | 8 hours | 4 sessions, 1-hour each  total = 4hours |
| Mode of delivery | Face to face workshop | Online zoom workshops due to COVID pandemic. |
| Additional preparations | undetermined  Not included in IECD method | Workshop materials sent to participants 3 weeks before workshop (Spreadsheet with AAA data element utilisation rates; policy review summary)  Online ECD data elements survey completed by participants before workshop 2 |
| Quantitative data presented at workshop  ● Data element utilization  rates  ● ECD data elements  online survey | Yes  Not applicable | Yes  Yes |
| Focus of workshop discussions for data elements | All data elements | Prioritised by results of ECD data elements online survey |
| Visual presentations during workshop | Screenshots of Powerform displayed on-screen during discussions  Use of tick, cross, arrow, question mark for workshop recommendations (keep, remove, move, undetermined) | Screenshots of Powerform with data element utilisation rates displayed on-screen throughout discussions  Use of tick, cross, arrow, question mark for workshop recommendations (keep, remove, move, undetermined) |
| **Phase 5**  ***Post Workshop debriefs*** |  |  |
| Focus | Not applicable | ECD team consolidation of recommendations, including data elements not discussed during workshop due to time constraints |

**APPENDIX B**

**Table of relevant policies for the AAA form**

| **Policies with specific relevance to AAA data elements** (satisfied policy review criteria) | | | | |
| --- | --- | --- | --- | --- |
| **Document** | **Clinical topic** | **Year last reviewed** | **Policy Level** | **AAA section with relevant data elements** |
| NSQHS Standard 6: Communicating for Safety | Healthcare documentation | 2017 | national | all |
| NSQHS Standard 5: Comprehensive Care | Integrated Care | 2017 | national | all |
| NSQHS Standard 4: Medication Safety Standard | Medication safety | 2017 | national | 1-Admission Risk Assessment Screen; 4-Screening for Risk |
| NSQHS Standard 5: Patient Identification and Procedure Matching | Patient Identification | 2012 | national | 1-Admission Risk Assessment Screen |
| NSW Health Protecting People and Property Manual | People and Property | 2018 | state | 12-19 Patient Belongings & Valuables |
| NSW Health PD2017_041: Nutrition Care | Nutrition | 2017 | state | 4-Screening for Risk, 6-Nutrition and Toileting |
| NSW Health PD2013_043: Medication Handling in NSW Public Health Facilities | Medications | 2013 | state | 1-Admission Risk Assessment Screen; 4-Screening for Risk |
| NSW Health PD2011_015: Care Coordination: Planning from Admission to Transfer of Care in NSW Public Hospitals | Transfer of care | 2011 | state | 11-Discharge Risk Assessment |
| **Other policies** (related but did not satisfy policy review criteria) | | | | |
| **Document** | **Clinical Topic** | **Year last reviewed** | **Policy Level** | **AAA section with relevant data elements** |
| NSW Health GL2008_001 Nursing & Midwifery Clinical Guidelines - Identifying & Responding to Drug & Alcohol Issues | Drug and Alcohol | 2008 | state | 4-Screening for Risk |
| Safe and high-quality care for patients with cognitive impairment (dementia and delirium) in hospital | Dementia | 2014 | national | 4-Screening for Risk, 7-Mental Status and Medications, 8-Abbreviated Mental Health Test |
| SLHD Delirium Policy – SLHD_PCP2019_023 | Delirium | 2019 | district | 4-Screening for Risk; 7-Mental Status and Medications, 8-Abbreviated Mental Health Test |
| SLHD Pressure Injury Prevention and Management Policy SLHD_PD2017_034 | Skin Integrity | 2017 | district | 4-Screening for Risk, 6-Nutrition and Toileting, 10-Waterlow Prevention Equipment V2 |

**APPENDIX C**

**Sample of pre-workshop data elements survey**

Section: Admission Risk Assessment Screen

*Data Element 1*

- Keep
- Move
- Remove

Comments/suggestions

*Data Element 2*

- Keep
- Move
- Remove

Comments/suggestions

*Data Element x*

- Keep
- Move
- Remove

Comments/suggestions
